# Post-quadri-vaccinal SARS-CoV-2 antibodies in Vietnamese patients with end-stage renal disease: A prospective cohort study

**DOI:** 10.1097/MD.0000000000046311

**Published:** 2026-05-12

**Authors:** Nghia Nhu Nguyen, Giau Minh Huynh, Tan Huynh Ngoc Mai

**Affiliations:** a Faculty of Medicine, Can Tho University of Medicine and Pharmacy, Can Tho, Vietnam; b Giong Rieng General Hospital, Can Tho, Vietnam.

**Keywords:** COVID-19 vaccine, end-stage renal disease, SARS-CoV-2 antibodies, quadri-vaccinal, Vietnam

## Abstract

Patients with chronic kidney disease are at high risk for severe coronavirus disease 2019 (COVID-19). Although vaccination offers protection, antibody titers in this population decline over time. This study measured SARS-CoV-2 spike protein IgG (anti-S) levels at 1 and 6 months after the 4th vaccine dose and identified factors associated with antibody decline in Vietnamese patients with end-stage renal disease receiving hemodialysis. A prospective cohort study was conducted on 87 end-stage renal disease patients undergoing hemodialysis at Can Tho General Hospital, Vietnam, after 4th dose of COVID-19 vaccine, from July 2022 to January 2023. The median anti-spike protein antibody (IgG anti-S) levels at 1 month (T1) after the 4th dose of COVID-19 vaccine in patients was 13559.0 AU/mL (7713.0–19608.0). After 6 months (T6), antibody levels decreased to 8197.0 AU/mL (4116.0–13793.0). Patients with multiple comorbidities and prolonged dialysis vintage were independent factors associated with lower antibody levels 6 months after the 4th dose of COVID-19 vaccine. All patients in our study had an immune response that produced IgG anti-S antibodies after receiving 4 doses of the COVID-19 vaccine. Median IgG anti-S concentration at 1 month was 13559.0 AU/mL (7713.0–19608.0), at 6 month was 8197.0 AU/mL (4116.0–13793.0). The more comorbidities and prolonged dialysis vintage the patients had, the lower the IgG anti-S concentration after 6 months.

## 1. Introduction

The coronavirus disease 2019 (COVID-19) pandemic was first diagnosed in Wuhan, China, as a result of SARS-CoV-2 infection. It quickly spread into a global pandemic, greatly impacting public health worldwide.^[1]^ Patients with chronic kidney disease (CKD) at any stage are more likely to have a poor prognosis when infected with SARS-CoV-2 because of the presence of multiple chronic diseases, such as hypertension, diabetes, and heart failure, which can worsen the disease.^[2,3]^ COVID-19 vaccination is a safe and reliable method to induce antibodies against SARS-CoV-2, helping reduce the incidence and severity of the disease. Several studies have shown that the Covid-19 vaccination can effectively produce antibodies against SARS-CoV-2. However, these antibodies decline over time, reducing their protective effect, especially in older patients with multiple comorbidities.^[4]^

Given this vulnerability, many countries have prioritized booster vaccination for high-risk groups, including patients with CKD. On July 22, 2022, the Vietnamese Ministry of Health launched a national campaign to strengthen COVID-19 vaccination, recommending the 4th dose for such populations. Hospitals across Vietnam subsequently administered the 4th dose to CKD patients, including those receiving maintenance hemodialysis. Despite these efforts, data on the kinetics of vaccine-induced antibodies in the Vietnamese CKD population remain scarce. Understanding the persistence and determinants of antibody levels after booster doses is essential to optimize vaccination strategies and timing.

Therefore, we conducted this study to compare SARS-CoV-2 spike protein antibody levels after 1 and 6 months of COVID-19 vaccination and to identify factors influencing the change in antibody levels after 6 months in Vietnamese patients with end-stage renal disease (ESRD) undergoing hemodialysis.

## 2. Materials and methods

### 2.1. Study design

A prospective cohort study was conducted on patients aged > 18 years with end-stage chronic kidney disease (according to KDIGO 2012) undergoing hemodialysis at Can Tho General Hospital, a large hospital in the Mekong Delta region, Vietnam. Both SARS-CoV-2 infected and uninfected patients were included in the study. The study was conducted from July 2022 to January 2023 with a 6-month follow-up period.

Patients were excluded from the study if they had any of the following conditions: current use of immunosuppressive drugs, such as corticosteroids, cyclophosphamide, cyclosporin A, mycophenolate mofetil, noncompliance with the study, or death or transfer to another treatment facility during the study period.

A total of 87 patients agreed to participate in the study, and all were vaccinated with the mRNA vaccine (BNT162b2, Pfizer-BioNTech) in accordance with the hospital’s vaccine policy.

Patients self-answered a questionnaire including information such as name, age, sex, underlying medical conditions, time on hemodialysis, and whether they had been previously infected with COVID-19. The quantitative assay for anti-spike protein antibody (IgG anti-S) concentration of SARS-CoV-2 virus was performed using an automated chemiluminescent immunoassay on a Cobas e 601 immunoassay analyzer. The study participants were tested using the Elecsys Anti-SARS-CoV-2 S test. The immune response to antibody levels was determined as follows:

Immune response to SARS-CoV-2: antibody level ≥ 15 AU/mL.

No immune response: antibody test results < 12 AU/mL.

We tested for antibodies for the first time after 1 month and the second time after 6 months of the 4th dose of COVID-19 vaccine and compared the antibody levels between the 2 times. In addition, we also investigated some factors related to antibody levels, such as age, sex, medical history, number of comorbidities, and time on dialysis.

### 2.2. Data analysis

Data were analyzed using the SPSS software (version 22.0; IBM Corp.).^[5]^ Descriptive statistics were used, including frequency and percentage for categorical variables, mean and standard deviation for quantitative variables with a normal distribution, and median and interquartile range for variables with a non-normal distribution. Some modifications must be made to enhance the readability and understanding of the text. Spearman correlation coefficient (*r*) was used to compare 2 continuous variables that did not follow a normal distribution.^[6]^ A positive value of *r* indicates a positive correlation, whereas a negative value indicates a negative one. The strength of the correlation is interpreted as follows^[6]^:

Very strong correlation: |*r*| ≥ .7

Moderate correlation: .3 ≤ |*r*| < .7

Weak correlation: |*r*| < .3

Multivariate linear regression analysis was performed to identify independent predictors of IgG anti-S antibody levels.

### 2.3. Research ethics

This study did not interfere with patient treatment. All the patients were fully informed and consented to participate in the study. The study was approved by the Ethics Committee of Can Tho University of Medicine and Pharmacy on July 26, 2022 (code number: 22.056), HV/PCT.HDDD.

## 3. Results

Table 1 presents the baseline characteristics of the participants. The mean age was 53.5 years, with most patients younger than 60, and 48.3% were male. The mean body mass index was 22.9 kg/m². The median dialysis vintage was 6.5 years, and all patients had at least 1 comorbidity, most commonly hypertension, followed by diabetes and cardiovascular disease. Laboratory results indicated advanced renal failure with mean estimated glomerular filtration rate <5 mL/min/1.73 m^2^.

**Table 1:** Patient characteristics.

Characteristics		Results
Male (%)		42 (48.3)
Age (yr)		53.48 (12.89)
Age group	<60	54 (62.1)
	≥60	33 (37.9)
BMI (kg/m^2^)		22.88 (3.74)
Dialysis vintage (yr)		6.5 (3.75)
Number of comorbidities	1	30 (34.5)
	2	38 (43.7)
	≥3	19 (21.8)
Hypertension (%)		29 (33.3)
Diabetes (%)		87 (100)
Cardiovascular disease (%)		18 (20.7)
Hepatitis B (%)		10 (11.5)
Hepatitis C (%)		6 (6.9)
Smoke (%)		22 (25.3)
Drink (%)		22 (25.3)
Urea (mmol/L)		26.30 ± 5.80
Creatinin (µmol/L)		1119.86 ± 272.0
eGFR (mL/min/1.73 m^2^)		4.34 ± 1.39

Data are shown as n (%) or mean ± SD.

BMI = body mass index, eGFR = estimated glomerular filtration rate.

**Figure 1.**
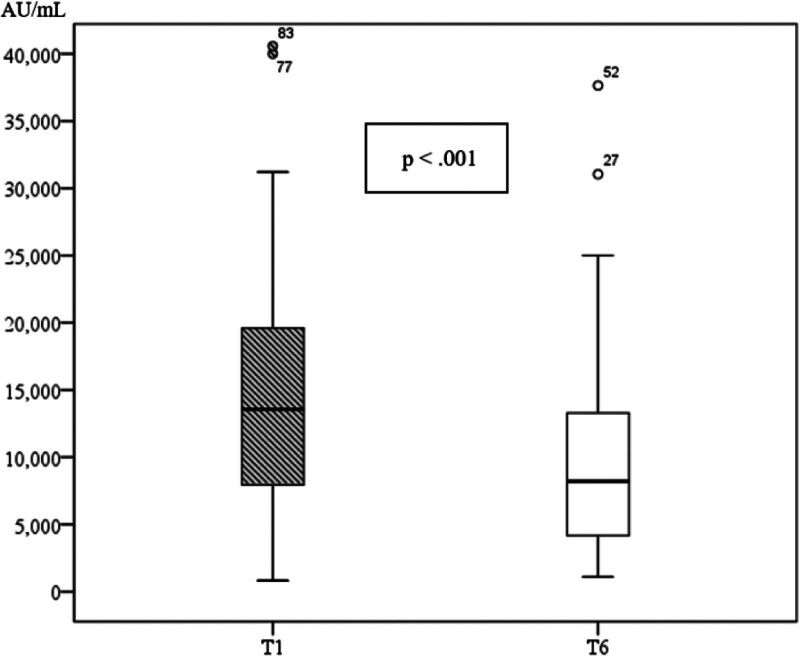
Anti-S IgG concentration at 1 month (T1) and 6 months (T6).

Figure 1 shows a significant decline in median anti-S IgG concentration from 1 month (T1) to 6 months (T6) after the 4th COVID-19 vaccine dose.

As shown in Table 2, anti-S IgG concentration was negatively correlated with age and the number of comorbidities and positively correlated with dialysis vintage and serum creatinine. No significant associations were found with body mass index, albumin, protein, or urea.

**Table 2:** Correlation between anti-S IgG concentration after 6 months and some characteristics of the patients.

Index	*r*	*P*-value	Equation
Age	‐.34	.001	*y* = 21232.583 – 214.433*x*
BMI	‐.02	.83	
Dialysis vintage	.29	.007	*y* = 5761.191 + 615.831*x*
Number of comorbidities	‐.66	.001	*y* = 18463.4 – 4586.908*x*
Albumin	.14	.21	
Protein	.19	.07	
Urea	.07	.53	
Creatinin	.31	.003	*y* = 1320.324 + 7.54*x*
eGFR	‐.23	.03	*y* = 14482.726 – 1087.448*x*

**Figure 2.**
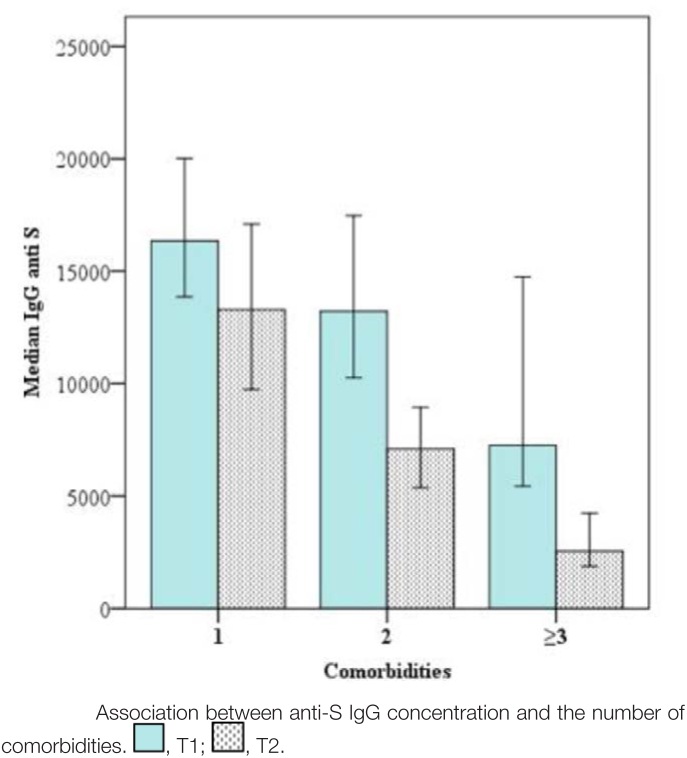


Figure 2 demonstrates that patients with fewer comorbidities had higher anti-S IgG levels at both T1 and T6.

In multivariate regression analysis, the number of comorbidities and hemodialysis vintage were independent factors affecting the antibody concentration of patients after 6 months (Table 3).

**Table 3:** Multivariate regression analysis of factors affecting antibody concentration after 6 months.

Parameter	Beta coefficients	95% confidence interval	*P*-value
Age	‐100.001	‐214.11 to 14.11	.085
Smoke	529.260	‐2601.66 to 3660.18	.737
No. of comorbidities	‐3905.930	‐6006.69 to ‐1805.17	.001
Dialysis vintage	430.902	43.03 to 818.77	.030
Diabetes	‐107.801	‐3898.02 to 3682.41	.955

## 4. Discussion

Several studies have investigated antibody levels and their changes after COVID-19 vaccination in patients with ESRD undergoing hemodialysis. However, to our knowledge, this is the first study in Vietnam with a follow-up period of up to 6 months to investigate antibody levels after 4 vaccine doses in patients with ESRD on hemodialysis (HD). Our patients had a relatively long dialysis duration, with an average of 6.5 years. All CKD patients in this study had at least 1 comorbidity, 43.7% had 2, and 21.8% had at least 3 comorbidities. Therefore, in addition to ESRD, most patients also have many other concurrent diseases that require treatment. The more comorbidities a patient has, the more it will affect their health status, including immune function.

There are various preventive measures against COVID-19, including vaccination, hand hygiene, social distancing, and quarantine.^[7,8]^ A study by Tran Van De et al reported that the majority of Vietnamese people used traditional medicines for COVID-19 prevention and treatment.^[9]^ However, vaccination is the most effective preventive measure.^[10]^ The Vietnamese Ministry of Health has issued a circular on strengthening COVID-19 vaccination for high-risk groups, including patients with ESRD. All patients in the study were vaccinated with the comirnaty mRNA vaccine from Pfizer/BioNTech (BNT162b2). One month (T1) after the 4th dose, we performed a quantitative assay for anti-spike protein antibodies against SARS-CoV-2 in patients using chemiluminescent immunoassay. After 6 months, we performed a second IgG anti-S concentration test. Our results showed that 100% of patients had developed antibodies after the 4th dose and the antibody levels peaked at 1 month and declined significantly by 6 months. Daisuke Kanai concluded that the HD patients group had significantly lower antibody concentrations at month 1 compared to the control group, and these concentrations remained lower at month 6 (CI 95%): 2617.1 (1296.7, 5240.8) versus 7285.4 (4403.9, 11000.0) AU/mL at month 1 and 353.4 (178.4, 656.3) versus 812.0 (498.3, 1342.7) AU/mL at month 6 (*P* < .001, respectively).^[11]^

Among the COVID-19 vaccines licensed for use in Vietnam by the Ministry of Health, Comirnaty by Pfizer/BioNTech (BNT162b2) is widely used, especially for booster shots.^[12]^ Martin et al compared the immunogenicity and clinical efficacy of the BNT162b2 (Pfizer) and ChAdOx1 (AstraZeneca) vaccines in HD patients and concluded that the mean IgG anti-S were higher in the BNT162b2 group than in the ChAdOx1 group, with antibody concentrations of 462 (152–1171) and 78 (20–213) BAU/mL (*P* < .001).^[2]^ Patrick Affeldt also concluded that antibody concentrations increased gradually after the 3rd and 4th doses, corresponding to 2882.7 (3093.0) and 6923.5 (8661.2) AU/mL, respectively. The authors also emphasized that booster vaccination for vulnerable groups is an important strategy to overcome the waning immune system, especially against emerging SARS-CoV-2 variants.^[13]^

Canetti et al, who studied the immunogenicity of the 4th dose of the COVID-19 vaccine, also found that antibody levels peaked 2 to 3 weeks after vaccination and then gradually declined. In the first month after vaccination, anti-RBD IgG antibody titers increased 9- to 10-fold. After 90 days, antibody levels decreased to 1442 BAU/mL (CI 95%: 1194–1741) in the Moderna vaccine group and 854 BAU/mL (CI 95%: 738–989) in the Pfizer vaccine group. After analysis, the authors found that patients’ IgG antibody levels decreased by approximately 2% per week over time.^[14]^ A study by Jan-Stephan F Sanders et al evaluated the anti-COVID-19 antibody levels after mRNA vaccination in CKD patients. The results showed that 6 months after vaccination, IgG anti-S antibodies of SARS-CoV-2 were present in 100% of the control group, 98.7% of CKD stage 4/5 patients, 95.1% of HD patients, and 56.6% of kidney transplant recipients. After 28 days, the authors noted a significant decrease in the antibody levels. The authors estimated the half-life of antibody levels in the blood of all 4 study groups to be 52 days (41–69). The authors concluded that measures to enhance the immune response, such as booster vaccination, are needed, especially in kidney transplant recipients.^[15]^

Our study is consistent with other domestic and international studies. The level of anti-COVID-19 antibodies was not constant but decreased over time.^[16]^ This was observed in both healthy individuals and CKD patients. However, according to foreign authors, the rate of decrease in antibody levels in CKD patients, especially kidney transplant recipients, was faster than that in healthy people. Therefore, it is necessary to investigate the risk factors that cause a rapid decrease in antibody levels in patients in order to develop timely measures to protect them. Besides, retesting for antibodies after vaccination is also very important, as it is the basis for deciding whether or not to revaccinate with the COVID-19 vaccine.

In this study, we investigated common comorbidities in patients with ESRD, including diabetes mellitus, hypertension, heart failure, chronic hepatitis B virus, and chronic hepatitis C virus. When analyzing the correlation between each comorbidity and IgG anti-S levels, we found no significant difference between the group with comorbidities and without. However, when analyzing the number of comorbidities per patient, we found that patients with multiple comorbidities had lower IgG anti-S levels than those with fewer comorbidities. This is easily explained by the fact that the more comorbidities a patient has, the more it will affect their health status, including immune function.^[17,18]^ Therefore, patients with multiple comorbidities need to be more closely monitored and cared for, and their antibody levels after COVID-19 vaccination need to be monitored to develop an appropriate revaccination plan to help increase antibody levels and protect patients.

A study by Antonella Zizza et al found that the rate of COVID-19 pneumonia was higher in the group with ≥ 3 comorbidities than in the group without comorbidities.^[19]^ Khong conducted a meta-analysis on the issue of the 4th dose of COVID-19 vaccine and concluded that the 4th dose should be considered for specific groups of individuals, especially the elderly, those with immunosuppression (such as those taking immunosuppressive drugs, kidney transplant recipients, etc), and those with chronic diseases (such as chronic kidney disease). The author also emphasized that the timing of the 4th dose is very important and should be individualized. Based on evidence from other studies, the author suggests that an interval of 4 months after the 3rd dose is appropriate, after which antibody levels start to decline significantly.^[20]^ This was also similar to the finding in our study, in which antibody levels in patients with CKD decreased by nearly 50% after 6 months. Most importantly, the author concluded that the importance of vaccines should never be overlooked, as new viral variants can emerge at any time.

Our study has some limitations, such as the inability to investigate IgG anti-S antibody levels after the 1st, 2nd, and 3rd doses of the vaccine; therefore, it was not possible to compare the antibody levels after each vaccination. However, T-cell immunity has not yet been evaluated. However, our study showed that 100% of patients after the 4th dose achieved high IgG anti-S antibody levels, suggesting that the COVID-19 vaccine booster was effective and necessary. In addition, due to the new regulations on COVID-19 diagnostic testing, and as COVID-19 has gradually become more “familiar,” the symptoms of the disease were also milder, so patients were not routinely tested to diagnose whether they had reinfected with the virus after vaccination. In particular, it was impossible to identify the viral strain if the patient was infected. Therefore, it was not possible to accurately assess the protective efficacy of antibodies against new SARS-CoV-2 variants in the future, as well as the impact of previous viral infection on antibody response in patients. All patients in the study were only vaccinated with the same mRNA vaccine, Pfizer, so it was also not possible to compare the antibody production efficiency and antibody level changes between different vaccines, as in other foreign studies.

## 5. Conclusion

All patients developed an immune response with detectable IgG anti-S antibodies after receiving 4 doses of the COVID-19 vaccine. Antibody levels peaked at 1 month and declined significantly by 6 months. A greater number of comorbidities and longer dialysis vintage were associated with lower IgG anti-S concentrations at 6 months.

## Acknowledgments

We would like to thank the Rectorate Board of the Cantho University of Medicine and Pharmacy and the Board of Directors of the Cantho General Hospital for creating favorable conditions for this study.

## Author contributions

**Conceptualization**: Nghia Nhu Nguyen.

**Data curation**: Tan Huynh Ngoc Mai, Giau Minh Huynh.

**Formal analysis**: Tan Huynh Ngoc Mai, Nghia Nhu Nguyen.

**Investigation**: Tan Huynh Ngoc Mai, Nghia Nhu Nguyen, Giau Minh Huynh.

**Methodology**: Tan Huynh Ngoc Mai.

**Supervision**: Nghia Nhu Nguyen.

**Validation**: Nghia Nhu Nguyen.

**Visualization**: Tan Huynh Ngoc Mai, Nghia Nhu Nguyen, Giau Minh Huynh.

**Writing – original draft**: Tan Huynh Ngoc Mai, Nghia Nhu Nguyen.

**Writing – review & editing**: Tan Huynh Ngoc Mai, Nghia Nhu Nguyen, Giau Minh Huynh.
